# Supplementary material for: Identification of Clinical and Genomic Features Associated with SARS-CoV-2 Reinfections
Source: Viruses. 2025 Jun 11;17(6):840. doi: 10.3390/v17060840 (PMC12197629; doi:10.3390/v17060840)
Supplement: Supplementary file 1 [file viruses-17-00840-s001.zip › viruses-3665803-supplementary.pdf]

Supplementary figures

Identification of clinical and genomic features associated with SARS-CoV-2 reinfections

Francisco Muñoz-López, Antoni E Bordoy, Francesc Català-Moll, Verónica Saludes, David Panisello Yagüe, Mariona Parera, Ignacio Blanco, Pere-Joan Cardona, Cristina Casañ, Ana Blanco-Suárez, Sandra Franco1, Álvaro F. García-Jiménez, Roger Paredes, Bonaventura Clotet, Lourdes Mateu, Marc Noguera-Julian, Elisa Martró, José Ramón Santos*, Marta Massanella*

Supplementary Figure 1

a

Event	Code
PCR/TMA+	A
PCR/TMA-	B
PCR sequenced	C
PCR potential sequencing	D
Antigen+	E
Antigen-	F
Serology+	G
Serology-	H
Vaccination - BNT162b2	I
Vaccination - mRNA-1273	J
Vaccination - AZD1222	K
Vaccination - Ad26.CoV2.S	L
Vaccination - Other	M
Hospital entry (probable COVID-19)	N
Hospital entry (possible COVID-19)	O
Hospital entry (ischemic embolic phenomena possibly derived from COVID-19)	P
Hospital discharge	Q
ICU/RICU entry	R
ICU/RICU discharge	S
Decease	T
Registered symptomatology	U

b

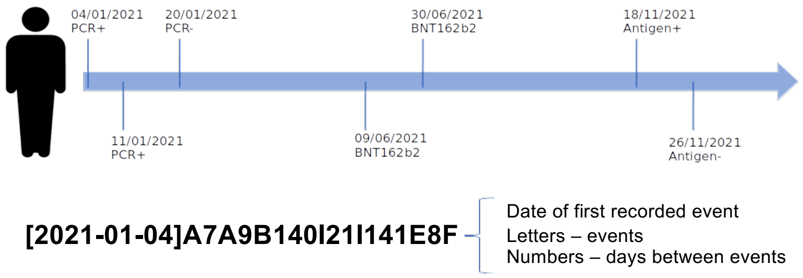

Supplementary Figure 1. Clinical timeline coding of the participants. (**a**) Table of significant events related to SARS-CoV-2 infections and vaccination. (**b**) Example of coding of the clinical chronology of a participant. The code starts with the date of the first registered event, the letters refer to events as in table A, and the numbers between letters are the number of days between consecutive events.

Supplementary Figure 2

a

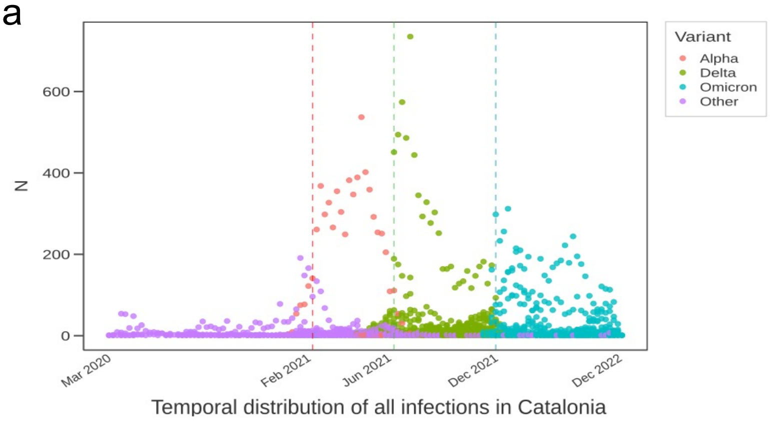

b

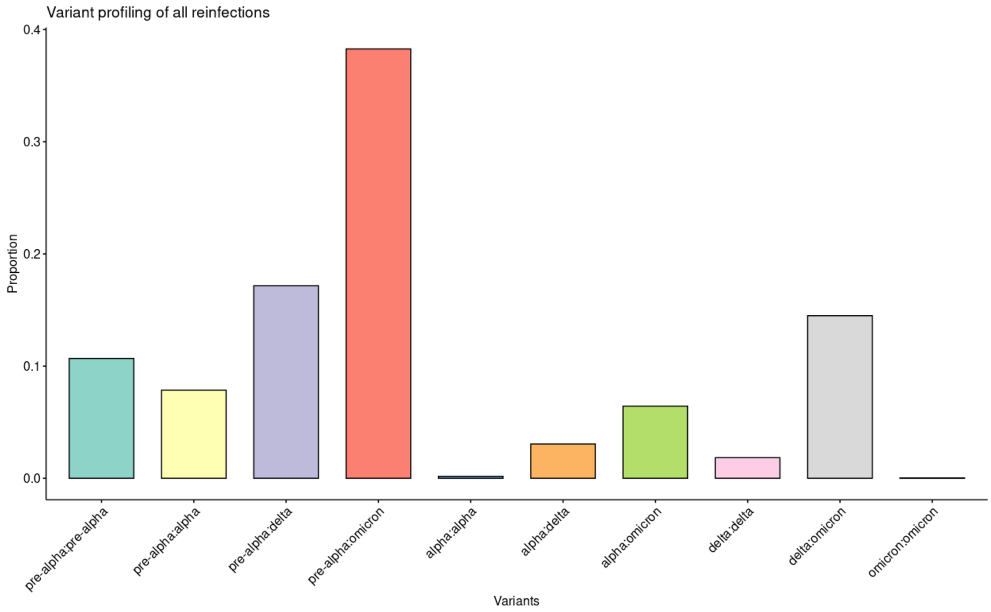

Supplementary Figure 2. SARS-CoV-2 variant estimations. (**a**) Temporal distribution of all SARS-CoV-2 infections in Catalonia from March 2020 to December 2022. Source: www.gisaid.org. (**b**) Estimation of the SARS-CoV-2 variants of the RECOVID Cohort following the temporal data given in (**a**).
